# Post-covid syndrome in individuals admitted to hospital with covid-19: retrospective cohort study

**DOI:** 10.1136/bmj.n693

**Published:** 2021-03-31

**Authors:** Daniel Ayoubkhani, Kamlesh Khunti, Vahé Nafilyan, Thomas Maddox, Ben Humberstone, Ian Diamond, Amitava Banerjee

**Affiliations:** 1 Office for National Statistics, Government Buildings, Newport, UK; 2 Diabetes Research Centre, University of Leicester, Leicester, UK; 3 Institute of Health Informatics, University College London, London NW1 2DA, UK; 4 University College London Hospitals NHS Trust, London, UK; 5 Barts Health NHS Trust, Royal London Hospital, London, UK

## Abstract

**Objective:**

To quantify rates of organ specific dysfunction in individuals with covid-19 after discharge from hospital compared with a matched control group from the general population.

**Design:**

Retrospective cohort study.

**Setting:**

NHS hospitals in England.

**Participants:**

47 780 individuals (mean age 65, 55% men) in hospital with covid-19 and discharged alive by 31 August 2020, exactly matched to controls from a pool of about 50 million people in England for personal and clinical characteristics from 10 years of electronic health records.

**Main outcome measures:**

Rates of hospital readmission (or any admission for controls), all cause mortality, and diagnoses of respiratory, cardiovascular, metabolic, kidney, and liver diseases until 30 September 2020. Variations in rate ratios by age, sex, and ethnicity.

**Results:**

Over a mean follow-up of 140 days, nearly a third of individuals who were discharged from hospital after acute covid-19 were readmitted (14 060 of 47 780) and more than 1 in 10 (5875) died after discharge, with these events occurring at rates four and eight times greater, respectively, than in the matched control group. Rates of respiratory disease (P<0.001), diabetes (P<0.001), and cardiovascular disease (P<0.001) were also significantly raised in patients with covid-19, with 770 (95% confidence interval 758 to 783), 127 (122 to 132), and 126 (121 to 131) diagnoses per 1000 person years, respectively. Rate ratios were greater for individuals aged less than 70 than for those aged 70 or older, and in ethnic minority groups compared with the white population, with the largest differences seen for respiratory disease (10.5 (95% confidence interval 9.7 to 11.4) for age less than 70 years *v* 4.6 (4.3 to 4.8) for age ≥70, and 11.4 (9.8 to 13.3) for non-white *v* 5.2 (5.0 to 5.5) for white individuals).

**Conclusions:**

Individuals discharged from hospital after covid-19 had increased rates of multiorgan dysfunction compared with the expected risk in the general population. The increase in risk was not confined to the elderly and was not uniform across ethnicities. The diagnosis, treatment, and prevention of post-covid syndrome requires integrated rather than organ or disease specific approaches, and urgent research is needed to establish the risk factors.

## Introduction

In the early stages of the covid-19 pandemic, the estimated infection rate of SARS-CoV-2 in the United Kingdom was 6% (13% in London).^1^ Research, health services, and the media have mostly focused on direct (through infection) and indirect (through changes in individual behaviours and health systems) effects of covid-19 on mortality,^2^ particularly in the short term.^3^ ^4^ Studies of the longer term effects on morbidity are needed to effectively plan healthcare delivery and capacity.

Since SARS-CoV-2 infection was recognised in late 2019, the academic and clinical emphasis has been on respiratory manifestations.^5^ Increasing evidence exists for direct multiorgan effects,^6^ ^7^ ^8^ ^9^ ^10^ however, and indirect effects on other organ systems and disease processes, such as cardiovascular diseases and cancers, through changes in healthcare delivery and patient behaviours.^11^ ^12^ ^13^ Although the long term effects of covid-19 on individuals and health systems are becoming clear, investigation across organ systems is urgently needed.

Long covid, or post-covid syndrome, is not one condition, and is defined by the National Institute for Health and Care Excellence (NICE) as “signs and symptoms that develop during or after an infection consistent with covid-19 which continue for more than 12 weeks and are not explained by an alternative diagnosis.”^14^ NICE guidelines recommend referral to post-covid syndrome assessment clinics if post-covid symptoms persist for 6-12 weeks.^14^ Pre-existing conditions and risk factors are predictors of acute covid-19 outcomes (such as admission to the intensive care unit and mortality^2^), but the epidemiology of post-covid syndrome has been less well defined^15^ ^16^ because of the unclear medium and long term pathophysiology across organ systems. When post-covid syndrome clinics are established, characterisation of the epidemiology of the disease will help with appropriate diagnosis, care, public health interventions and policy, and resource planning.

The existing evidence suggests large variations in estimates of the prevalence and incidence of post-covid syndrome because of the differences in study populations, recruitment methods, follow-up periods, and sample sizes. Most studies so far have focused on symptoms associated with post-covid syndrome rather than organ dysfunction, and few have made use of a control group, allowing the inference of counterfactual outcomes.

We aimed to estimate the excess morbidity after severe covid-19 disease, reflecting an urgent need for such evidence by policy makers. From national electronic health records and death registrations for individuals in England, we quantified the incidence of mortality, use of health services, and organ specific impairment in individuals with covid-19 after discharge from hospital. We estimated rate ratios for death, readmission, and multiorgan dysfunction after discharge from hospital compared with those in a matched general population control group, and the variations in the rate ratio (comparing outcome rates after hospital admission for covid-19 with expected risk in the general population) across demographic groups.

## Methods

### Study design and data sources

We conducted an observational, retrospective, matched cohort study of individuals admitted to hospital with covid-19. We used the Hospital Episode Statistics Admitted Patient Care^17^ records for England up to 31 August 2020 and the General Practice Extraction Service Data for Pandemic Planning and Research (GDPPR)^18^ up to 30 September 2020. GDPPR is an extract of primary care records collected from surgeries by NHS Digital for pandemic research and analysis (supported by the British Medical Association and the Royal College of General Practitioners), including over 56 million individuals registered at NHS England general practice surgeries and updated fortnightly. The extract includes a subset of about 35 000 clinical codes, selected for potential use in pandemic related analysis. Death registrations from the Office for National Statistics were linked for deaths up to 30 September 2020 and registered by 7 October 2020.

### Study population

Individuals were included if they had a hospital episode from 1 January to 31 August 2020 with a primary diagnosis of covid-19, identified with the International Classification of Diseases, 10th revision (ICD-10) codes U07.1 (virus identified) and U07.2 (virus not identified); that is, by a positive laboratory test or clinical diagnosis. We included patients coded as U07.2 because not all patients with covid-19 received a test during their hospital episode, particularly during the early weeks of the pandemic, recognising the role of clinical judgment. In sensitivity analyses, we included only patients diagnosed with code U07.1. Individuals with covid-19 were excluded if they were not discharged alive by 31 August 2020 or their date of birth or sex was not known. The index date was set to the date of discharge after the first hospital episode with covid-19 as the primary diagnosis.

Candidate controls were individuals in the general population who: did not meet the inclusion criteria for covid-19; had not died before 1 January 2020; and had at least one GDPPR record between 1 January 2019 (one year before the start of the follow-up period) and 30 September 2020 (end of the study). We applied the GDPPR criterion to ensure the controls were currently active patients within the NHS (eg, they had not emigrated without deregistering from their general practice). Each control had the same index date as their matched patient. We selected controls from the general population rather than matching to non-covid hospital admissions to determine the increased risk after hospital admission for covid-19 versus no hospital admission for covid-19 (that is, compared with the expected risk for people with similar personal and clinical characteristics in the general population).

### Outcome variables

Individuals were followed up from the index date to 30 September 2020 or the date of death (whichever was earlier) for all cause mortality, all cause hospital readmission (admission after discharge for patients and admission after the index date for controls), respiratory disease, major adverse cardiovascular event (a composite of heart failure, myocardial infarction, stroke, and arrhythmia), diabetes (type 1 or 2), chronic kidney disease stages 3-5 (including dialysis and kidney transplant), and chronic liver disease.

Diagnoses of respiratory disease, major adverse cardiovascular event, diabetes, chronic kidney disease, and chronic liver disease were identified from primary care and hospital records, except for the arrhythmia component of major adverse cardiovascular event for which primary care data were not available (although diagnoses made in hospital were recorded). The supplementary material contains the full code lists for the outcome variables.****

### Matching variables

We matched patients to controls on potential confounders of the relation between hospital admission for covid-19 and outcomes (supplementary table 1), established from electronic health records over a 10 year look back period (1 January 2010 to 31 December 2019). Personal factors recorded were age, sex, ethnicity, region, and deprivation. Comorbidities included the diagnoses listed above and hypertension and cancer, identified from diagnoses made in primary care and in hospital (with primary and secondary ICD-10 codes for the hospital diagnoses). We also included smoking status and body mass index in the matching set as risk factors. We broadly categorised age (<50, 50-69, ≥70) and body mass index (<25, 25 to <30, ≥30) to facilitate exact matching, which would not have been possible with continuous variables.

### Statistical techniques

Distributions for baseline characteristics were compared between individuals with covid-19 and a random 0.5% sample of the general population with χ^2^ tests and standardised differences in proportions, where a standardised difference or more than 10% indicated a large imbalance between groups.^19^ Patients were matched 1:1 to controls with coarsened exact matching,^20^ resulting in a perfect balance of joint distributions across the full range of (coarsened) variables included in the matching set, derived from 10 years of records. Matched pairs were discarded if the control died before the corresponding patient’s index date. All covariates were categorised before matching, including an unknown category comprising individuals with missing values. The size of the pool of candidate controls (about 50 million individuals) precluded the use of multiple imputation.

We computed rates of death, readmission, and multiorgan dysfunction after discharge from hospital per 1000 person years in patients and controls, deriving rate ratios from these rates. The 95% confidence intervals were estimated with the Poisson distribution. We estimated rates for all diagnoses (new onset diagnoses and exacerbation of pre-existing conditions) and only new onset diagnoses (that is, no previous diagnosis for the condition over the 10 year look back period). All rates were stratified by sex, age group (<70, ≥70), and ethnic group (white, non-white). The threshold of 70 years was chosen for age stratified analyses as the government of the United Kingdom has consistently stated that individuals aged 70 or more have a higher risk of severe illness from covid-19 (eg, in the government’s definition of the clinically vulnerable population in social distancing guidelines). Individuals with missing information for ethnicity were omitted from all analyses stratified by ethnic group. Patients were further stratified based on whether they were admitted to an intensive care unit during their hospital stay.

Sensitivity analysis investigated possible residual confounding by age, smoking status, and body mass index after matching because we had to use coarse versions of the variables to ensure a sufficient match rate. We assessed the robustness of our main results by adjusting for a second order polynomial of age and non-coarsened versions of smoking status and body mass index in a Poisson regression of outcome counts, including the natural logarithm of person years as an offset term. All statistical analyses were conducted with R version 4.0.2.

### Patient and public involvement

Although we did not directly involve patients and the public because of the covid-19 pandemic, views expressed by patient groups in meetings attended by DA, VN, and BH (eg, NHS England’s long covid taskforce, Department of Health and Social Care’s long covid round table) informed the study objectives and design.

## Results

### Study participants

**Fig 1:**
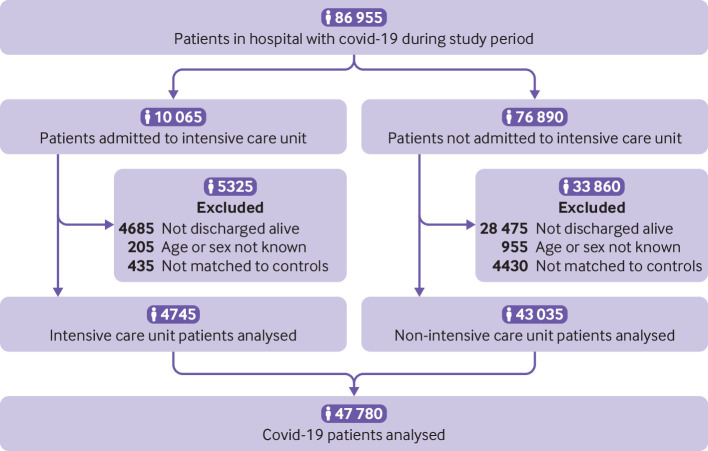
Study population flow diagram. Patient counts have been rounded to the nearest five for disclosure control reasons, and components may therefore not sum to totals

Of 86 955 individuals in hospital with covid-19 during the study period, 53 795 (61.9%) had been discharged alive by the end of the study (fig 1). After excluding individuals whose age or sex was not known and those who could not be matched to a control, 47 780 patients with covid-19 (4745 admitted to the intensive care unit and 43 035 not requiring admission to the intensive care unit) were included in the analysis, representing 90.8% of those discharged alive with known age and sex. Mean follow-up was 140 days (standard deviation 50 days, maximum 253 days) for patients with covid-19 and 153 days (33 days, 253 days) for controls.

At baseline, individuals with covid-19 had a mean age of 64.5 (standard deviation 19.2) and 54.9% were men. Compared with the general population, individuals in hospital with covid-19 were more likely to be: male, aged 50 or more, living in a deprived area, a former smoker, and overweight or obese (table 1). Individuals with covid-19 were also more likely to be comorbid than the general population, with a higher prevalence of previous admission to hospital and of all measured pre-existing conditions (most notably hypertension, major adverse cardiovascular event, respiratory disease, and diabetes).

**Table 1:** Baseline characteristics of individuals in hospital with covid-19 in England compared with those of a random sample from the general population and in the matched control group

Characteristic and category	Patients with covid-19 (n=47 780; sample distribution, No (%))	General population, before matching (n=239 380)			Matched control group, after matching (n=47 780)	
		Sample distribution (No (%))	Standardised difference *v* patients (%)		Sample distribution (No (%))	Standardised difference *v* patients (%)
Age						
<30	2255 (4.7)	75 700 (31.6)	−74.5		1190 (2.5)	11.9
30-49	7760 (16.2)	61 430 (25.7)	−23.3		8820 (18.5)	−5.9
50-69	15 945 (33.4)	61 500 (25.7)	16.9		15 945 (33.4)	0.0
≥70	21 825 (45.7)	35 715 (14.9)	71.0		21 825 (45.7)	0.0
Sex						
Men	26 245 (54.9)	107 890 (45.1)	19.8		26 245 (54.9%)	0.0
Women	21 535 (45.1)	126 450 (52.8)	−15.5		21 535 (45.1%)	0.0
Ethnicity						
White	34 355 (71.9)	151 180 (63.2)	18.8		34 355 (71.9%)	0.0
Asian	4320 (9.0)	15 150 (6.3)	10.2		4320 (9.0%)	0.0
Black	2565 (5.4)	6840 (2.9)	12.7		2565 (5.4%)	0.0
Mixed/other	1430 (3.0)	7010 (2.9)	0.4		1430 (3.0%)	0.0
Unknown	5110 (10.7)	59 205 (24.7)	−37.4		5110 (10.7%)	0.0
Index of Multiple Deprivation category						
1 (most deprived)	11 510 (24.1)	48 555 (20.3)	9.2		11 510 (24.1%)	0.0
2	10 970 (23.0)	47 680 (19.9)	7.4		10 970 (23.0%)	0.0
3	9265 (19.4)	47 125 (19.7)	−0.8		9265 (19.4%)	0.0
4	8315 (17.4)	46 040 (19.2)	−4.7		8315 (17.4%)	0.0
5 (least deprived)	7695 (16.1)	44 795 (18.7)	−6.9		7695 (16.1%)	0.0
Unknown	25 (<0.1)	5185 (2.2)	−20.3		25 (<0.1%)	0.0
Smoking status						
Current	4000 (8.4)	38 040 (15.9)	−23.2		4000 (8.4%)	0.0
Former	19 560 (40.9)	56 210 (23.5)	38.0		19 560 (40.9%)	0.0
Never	20 295 (42.5)	93 750 (39.2)	6.7		22 000 (46.0%)	−7.2
Unknown	3920 (8.2)	51 375 (21.5)	−38.0		2215 (4.6%)	14.6
Body mass index						
<25	9415 (19.7)	60 140 (25.1)	−13.0		12 345 (25.8%)	−14.7
25 to <30	12 140 (25.4)	48 290 (20.2)	12.5		12 140 (25.4%)	0.0
≥30	15 390 (32.2)	40 795 (17.0)	35.8		15 390 (32.2%)	0.0
Unknown	10 835 (22.7)	90 155 (37.7)	−33.1		7905 (16.5%)	15.5
Previous admission to hospital	39 575 (82.8)	150 510 (62.9)	46.0		37 930 (79.4)	8.8
Hypertension	24 720 (51.7)	43 170 (18.0)	75.6		24 720 (51.7)	0.0
Respiratory disease	19 440 (40.7)	38 695 (16.2)	56.5		19 440 (40.7)	0.0
Asthma	8695 (18.2)	27 345 (11.4)	19.2		9270 (19.4)	−3.1
COPD	6565 (13.7)	7090 (3.0)	39.7		5900 (12.4)	4.1
Other	11 890 (24.9)	20 405 (8.5)	45.0		10 124 (21.2)	8.8
Diabetes	11 680 (24.4)	16 670 (7.0)	49.5		11 680 (24.4)	0.0
Type 1	1235 (2.6)	1770 (0.7)	14.5		920 (1.9)	4.4
Type 2	11 530 (24.1)	15 810 (6.6)	50.1		11 475 (24.0)	0.3
MACE	11 650 (24.4)	13 385 (5.6)	54.6		11 650 (24.4)	0.0
Heart failure	5255 (11.0)	4150 (1.7)	38.7		4595 (9.6)	4.5
Stroke	3040 (6.4)	3100 (1.3)	26.6		2580 (5.4)	4.1
Myocardial infarction	2265 (4.7)	3160 (1.3)	20.1		2635 (5.5)	−3.5
Arrhythmia	7170 (15.0)	7540 (3.1)	42.2		7060 (14.8)	0.6
Cancer	9820 (20.5)	22 090 (9.2)	32.2		9820 (20.5)	0.0
Chronic kidney disease stages 3-5	6075 (12.7)	7930 (3.3)	35.1		6075 (12.7)	0.0
Chronic liver disease	1380 (2.9)	3005 (1.3)	11.5		1380 (2.9)	0.0

COPD=chronic obstructive pulmonary disease; MACE=major adverse cardiovascular event.

Baseline characteristics extracted from primary care records and hospital admissions from 1 January 2010 to 31 December 2019. The general population is a random 0.5% sample of individuals with primary care records between 1 January 2019 and 30 September 2020. Patients with covid-19 were matched to controls for baseline personal characteristics (age, sex, ethnicity, region, Index of Multiple Deprivation category, and smoking status) and clinical histories (hypertension, major adverse cardiovascular event, respiratory disease, chronic kidney disease, chronic liver disease, diabetes, and cancer).

Standardised differences in baseline characteristics between patients and controls were generally below 10%, and most were zero because of the use of exact matching. Individuals aged less than 30 and those whose smoking status or body mass index, or both, were not known, were more common in patients than in controls (as we matched on coarsened versions of these variables). Sensitivity analyses investigating the effect of adjusting for these variables showed minimal change in estimated rate ratios of multiorgan dysfunction between patients and controls, even when stratified by personal characteristics, indicating the absence of residual confounding after matching (supplementary sensitivity analyses).

### Rates of death, readmission, and multiorgan dysfunction in individuals with covid-19 after discharge from hospital

Of 47 780 individuals in hospital with covid-19 over the study period, 29.4% were readmitted and 12.3% died after discharge (table 2). These events occurred at rates of 766 (95% confidence interval 753 to 779) readmissions and 320 (312 to 328) deaths per 1000 person years, which were 3.5 (3.4 to 3.6) and 7.7 (7.2 to 8.3) times greater, respectively, than those in matched controls. Respiratory disease was diagnosed in 14 140 individuals (29.6%) after discharge, with 6085 of these being new onset diagnoses; the resulting rates of 770 (95% confidence interval 758 to 783) and 539 (525 to 553) per 1000 person years, respectively, were 6.0 (5.7 to 6.2) and 27.3 (24.0 to 31.2) times greater than those in controls.

**Table 2:** Counts and rates of death, readmission, and respiratory disease in individuals with covid-19 in England discharged from hospital by 31 August 2020 compared with matched controls

Outcome (sample size per group)	Patients with covid-19			Control group	
	Events (No (%))	Rate per 1000 person years (95% CI)		Events (No (%))	Rate per 1000 person years (95% CI)
Death (n=47 780)	5875 (12.3%)	320.0 (311.9 to 328.3)		830 (1.7%)	41.3 (38.6 to 44.3)
Readmission to hospital (n=47 780)	14 060 (29.4%)	766.0 (753.4 to 778.8)		4385 (9.2%)	218.9 (212.4 to 225.4)
Respiratory disease (all events) (n=47 780)	14 140 (29.6%)	770.5 (757.8 to 783.3)		2585 (5.4%)	129.1 (124.2 to 134.2)
Respiratory disease (new onset) (n=28 335)	6085 (21.5%)	538.9 (525.5 to 552.6)		240 (0.8%)	19.7 (17.3 to 22.4)

Outcomes calculated from hospital admissions to 31 August 2020, and primary care records and deaths registrations to 30 September 2020. Readmission to hospital is any admission after discharge for patients with covid-19 and any admission after the index date for controls. Patients with covid-19 were matched to controls for baseline personal characteristics (age, sex, ethnicity, region, Index of Multiple Deprivation category, and smoking status) and clinical histories (hypertension, major adverse cardiovascular event, respiratory disease, chronic kidney disease, chronic liver disease, diabetes, and cancer).

**Fig 2:**
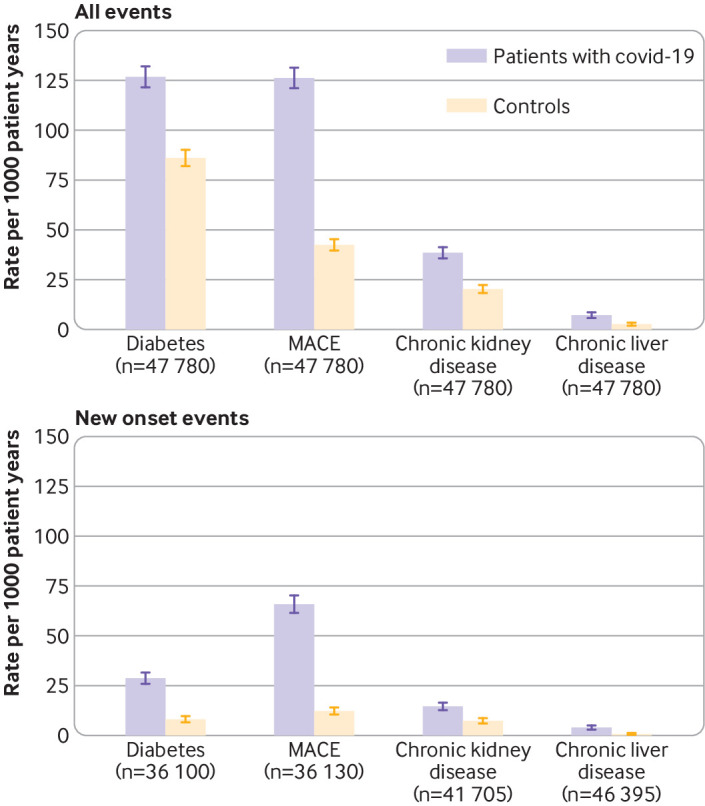
Rates of multiorgan dysfunction comparing individuals with covid-19 in England discharged from hospital by 31 August 2020 with matched controls. Outcomes calculated from hospital admissions to 31 August 2020, and primary care records and deaths registrations to 30 September 2020. Patients with covid-19 were matched to controls for baseline personal characteristics (age, sex, ethnicity, region, Index of Multiple Deprivation category, and smoking status) and clinical histories (hypertension, major adverse cardiovascular event, respiratory disease, chronic kidney disease, chronic liver disease, diabetes, and cancer). MACE=major adverse cardiovascular event

Diabetes, major adverse cardiovascular event, chronic kidney disease, and chronic liver disease were diagnosed after discharge in 4.9%, 4.8%, 1.5%, and 0.3% of individuals with covid-19, respectively, occurring at rates of 127 (122 to 132) for diabetes, 126 (121 to 131) for major adverse cardiovascular event, 39 (36 to 42) for chronic kidney disease, and 7 (6 to 9) for chronic liver disease diagnoses per 1000 person years (fig 2). We saw a similar pattern when only new onset diagnoses were considered, but at lower rates of 29 (26 to 32) for diabetes, 66 (62 to 70) for major adverse cardiovascular event, 15 (13 to 17) for chronic kidney disease and 4 (3 to 5) for chronic liver disease diagnoses per 1000 person years. Those with covid-19 were diagnosed with major adverse cardiovascular event, chronic liver disease, chronic kidney disease, and diabetes after discharge from hospital 3.0 (2.7 to 3.2), 2.8 (2.0 to 4.0), 1.9 (1.7 to 2.1), and 1.5 (1.4 to 1.6) times more frequently, respectively, than in the matched control group (supplementary table 2).

Rates of death, readmission, and multiorgan dysfunction after discharge from hospital remained substantially increased in individuals with covid-19 compared with matched controls, after stratifying by admission to the intensive care unit versus no admission to the intensive care unit (supplementary table 3). Individuals who needed to be admitted to the intensive care unit had higher rates of respiratory disease and diabetes after discharge, but lower rates of death, readmission, and major adverse cardiovascular event, than those who did not need to be admitted to the intensive care unit.

In sensitivity analyses, comparisons between outcome rates for patients and controls were robust when only laboratory confirmed diagnoses of covid-19 were included, representing 80.2% of all patients with covid-19 in the study. We also explored the robustness of our findings when 4865 patients with covid-19 (9.2%) that were unmatched, and therefore excluded from our main analysis, were added to the study population. We found that outcome rates in the matched population could have slightly underestimated the rates in the full population of patients with covid-19 who were discharged. The estimates presented in our main results could therefore be conservative (supplementary sensitivity analyses).

### Rate ratios of death, readmission, and multiorgan dysfunction after discharge across demographic characteristics

**Fig 3:**
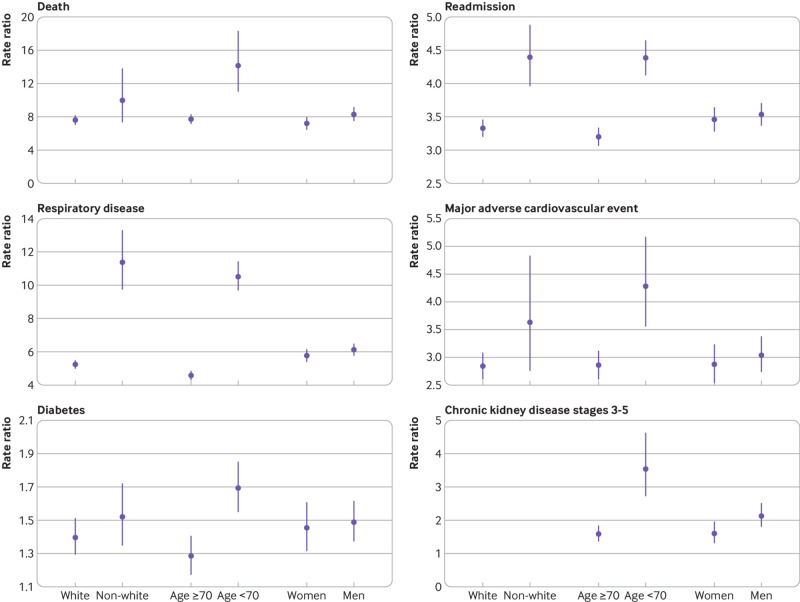
Rate ratios of death, readmission, and multiorgan dysfunction comparing individuals with covid-19 in England discharged from hospital by 31 August 2020 with matched controls, stratified by personal factors. Outcomes calculated from hospital admissions to 31 August 2020, and primary care records and deaths registrations to 30 September 2020. Readmission to hospital is any admission after discharge for patients with covid-19 and any admission after the index date for controls. Individuals with missing ethnicity information were omitted from the analysis stratified by ethnic group. Patients with covid-19 were matched to controls for baseline personal characteristics (age, sex, ethnicity, region, Index of Multiple Deprivation category, and smoking status) and clinical histories (hypertension, major adverse cardiovascular event, respiratory disease, chronic kidney disease, chronic liver disease, diabetes, and cancer). Rate ratios for chronic kidney disease could not be stratified by ethnic group because of insufficient event counts in the control group

Rates of all outcomes after discharge were greater in individuals with covid-19 aged 70 or more than in those aged less than 70, whereas rates of all outcomes other than diabetes were greater in the white ethnic group than in the non-white group (supplementary table 4). Rate ratios comparing patients with covid-19 and matched controls were greater in individuals aged less than 70 than those aged 70 or more for all outcomes, however (fig 3). The largest differences in rate ratios were for death (14.1 (95% confidence interval 11.0 to 18.3) for age <70 years *v* 7.7 (7.1 to 8.3) for ≥70) and respiratory disease (10.5 (9.7 to 11.4) for age <70 *v* 4.6 (4.3 to 4.8) for ≥70). Ethnic differences in rate ratios were greatest for respiratory disease (11.4 (9.8 to 13.3) for individuals in the non-white group *v* 5.2 (5.0 to 5.5) in the white ethnic group). Differences in rate ratios between men and women were generally small (supplementary table 4).

## Discussion

### Principal findings

Three major findings were found in this large study examining post-covid syndrome in 47 780 patients admitted to hospital with covid-19 in England, matched to controls. Firstly, admission to hospital for covid-19 was associated with an increased risk of readmission and death after discharge compared with individuals with similar personal and clinical characteristics in the general population over the same period. After admission to hospital for covid-19, 29% were readmitted and 12% died within a mean follow-up of 140 days. Secondly, rates of multiorgan dysfunction after discharge were raised in individuals with covid-19 compared with those in the matched control group, suggesting extrapulmonary pathophysiology. Diabetes and major adverse cardiovascular event were particularly common, whether incident or prevalent disease. Thirdly, the absolute risk of death, readmission, and multiorgan dysfunction after discharge was greater for individuals aged 70 or more than for those aged less than 70, and for individuals of white ethnic background than non-white individuals. Compared with outcome rates that might be expected to occur in these groups in the general population, however, younger patients and ethnic minority individuals had greater relative risks than those aged 70 or more and those in the white ethnic group, respectively.

In the secondary analysis, we found that individuals discharged from the intensive care unit after covid-19 experienced greater rates of death and readmission than those not admitted to the intensive care unit, perhaps because at risk covid-19 patients (whether based on age, multimorbidity, or irreversible causes of deterioration) were treated outside of the intensive care unit based on local protocols. Also, given the greater proportion of patients that did not need to be admitted to the intensive care unit versus those admitted to the intensive care unit who were discharged alive (63% *v* 53%), our results might reflect, at least in part, a survivorship effect.

### Comparison with related studies

Our results are consistent with proposed biological mechanisms associated with respiratory,^21^ cardiovascular,^22^ metabolic,^23^ renal,^10^ and hepatic^8^ involvement in covid-19, extending the early evidence base on post-covid syndrome which has been described as limited and of low quality.^24^

In a recent study of 1775 veterans in the United States admitted to hospital with covid-19, 20% were readmitted and 9% died within 60 days of discharge.^25^ After restricting follow-up in our study to the same length of time, we found similar prevalence rates of 23% and 9%, respectively. The US study did not analyse organ specific endpoints and was conducted in a specific population. Our study extends these findings as we found that covid-19 was associated with dysfunction in a range of organs after discharge in a broader population of patients admitted to hospital.

Multiorgan involvement after covid-19 was detected in 201 low risk individuals in the UK (18% admitted to hospital with covid-19), and impairment of the lungs (33%), heart (32%), kidneys (12%), and liver (10%) was common.^26^ These rates were higher than those estimated in our study, although organ dysfunction was mild and potentially subclinical. Among 213 individuals with covid-19 in the US who were discharged from hospital, 10% were readmitted and 2% died over a median follow-up of 80 days^27^ compared with our estimates of 29% and 12%, respectively (but with a longer median follow-up of 160 days). The small sample size precludes extrapolation to broader populations, however.

An association between covid-19 and an increased odds of acute kidney injury, renal replacement treatment, use of insulin, pulmonary embolism, stroke, myocarditis, arrythmia, and increased troponin was found in US veterans admitted to hospital with covid-19 versus a control group of patients with seasonal influenza.^28^ The index event was admission rather than discharge, so the results are not strictly comparable with our study, but suggest physiological changes in multiple organs after admission to hospital for covid-19, supporting our findings.

Pulmonary lesions were found in patients with covid-19 admitted to hospital in Wuhan, China, after a short follow-up of three weeks after discharge.^29^ Cardiovascular magnetic resonance imaging showed myocardial inflammation in German participants who recovered from acute covid-19,^6^ and myocarditis in US college athletes after acute covid-19 disease.^30^ These studies suggest pulmonary and myocardial involvement in individuals with covid-19 and, although small sample sizes and highly specific study populations make it difficult to generalise the results, they shed some light on possible pathophysiological mechanisms underlying our own findings.

Although we found that readmission occurred frequently for patients admitted to hospital for covid-19, we did not analyse the most common reasons for readmission. A US study of over 2000 patients admitted to hospital found that covid-19, sepsis, pneumonia, and heart failure were the most common reasons for readmission post-covid-19.^25^ Further research is needed, however, particularly the extent to which improvements in the management of post-covid syndrome (such as the recent NICE clinical guidelines^14^) might reduce readmission rates.

### Implications of our findings

With over three million people in the UK having tested positive for covid-19 at the time of writing,^31^ and many more who have had the disease but have never received a test, our findings suggest that the long term burden of covid-19 related morbidity on hospitals and broader healthcare systems might be substantial. Also, organ dysfunction in hospital patients represents only part of the problem; other symptomatic manifestations of post-covid syndrome in individuals not requiring admission to hospital have the potential to be debilitating for patients, placing a considerable burden on healthcare resources, particularly in primary care.

Post-covid syndrome adds to current healthcare challenges, particularly sustainable high quality care for long term conditions: inequalities in health, access, and provision; incomplete pathways across community and hospital care; and the need to translate research into clinical practice with sufficient resources. Our findings across organ systems suggest that the diagnosis, treatment, and prevention of post-covid syndrome requires integrated rather than organ or disease specific approaches. Integrated care pathways,^32^ effective in other diseases, such as chronic obstructive pulmonary disease, could be useful in the management of post-covid syndrome.

### Strengths and limitations

The strengths of our study include its size and completeness, with all individuals in England admitted to hospital with covid-19 observed over a follow-up period of several months, matched to general population controls from 10 years of clinical records. Like all observational studies, residual confounding is possible (eg, because of biomarkers or socioeconomic factors omitted from our matching set). Limited events in the control group meant we could not disaggregate rate ratios stratified by age and ethnicity beyond age less than 70 versus 70 or older and white versus non-white groups, despite likely variations in outcomes within these groups. Individuals with undiagnosed hypertension and diabetes were classified as not having these conditions as we did not consider blood pressure and measurements of glycated haemoglobin when defining matching variables. As a result of the Quality and Outcomes Framework (pay for performance), however, primary care coding for hypertension and diabetes is generally of high quality. Performing multiple imputation for missing values was not practical because of the size of the study dataset; instead we adopted the missing indicator approach, which could cause some bias in non-randomised studies.^33^ But the missing mechanism in clinical records might to some extent be “missing not at random” (eg, individuals who are neither underweight nor overweight could be less likely to have their body mass index measured), which would preclude the use of standard imputation techniques.

The hospital admission threshold might be lower in individuals with recent covid-19 disease than in the general population, and rates of diagnoses in general might have decreased indirectly because of the pandemic, particularly in people not admitted to hospital with covid-19. We could not access testing data so some individuals with covid-19 who did not require admission to hospital might have been matched in the control group. Also, our results are unlikely to fully capture the lived experiences of individuals with post-covid syndrome who were possibly asymptomatic and untested at the time of infection. Multiorgan post-covid manifestations have been identified in individuals not admitted to hospital,^26^ who were beyond the scope of our study. We did not capture symptoms such as fatigue, disturbances in taste and smell, and anxiety, widely reported in post-covid syndrome.^24^ Although we focused on outcomes after discharge for patients admitted to hospital for covid-19, a sizeable minority of individuals (38%) had not been discharged alive by the end of the study period, as reported globally.^34^ ^35^

### Choice of control group

We selected a matched control group from the general population of England, allowing estimation of the excess post-covid morbidity after severe covid-19 disease. An alternative approach might have involved comparing outcomes after covid-19 and other hospital admissions; such research has recently been conducted with similar data sources to those in our own study (although with a smaller covid-19 cohort), and comparable rates of organ dysfunction were found between patients with covid-19 and patients with pneumonia who were discharged from hospital in 2019.^36^ We believe that our study design, where comparisons were made with the expected risk in the general population, was more relevant to public health policy, and complementary to the study that used non-covid hospital admissions as the comparison group. Also, the use of non-covid hospital admissions as the comparison group does not allow estimation of excess morbidity because non-covid admission does not necessarily represent an appropriate counterfactual situation to admission to hospital for covid-19, and the size and direction of the inferences will depend on the choice of control admissions.

### Conclusions

Individuals discharged from hospital after acute covid-19 had an increased risk of mortality, readmission, and multiorgan dysfunction compared with similar individuals in the general population, and the relative increase in risk was not confined to the elderly and was not uniform across ethnic groups. Urgent research is needed to understand the risk factors for post-covid syndrome so that treatment can be targeted better to demographically and clinically at risk populations.

**What is already known on this topic**

Extrapulmonary dysfunction, affecting the cardiovascular, metabolic, renal, and hepatic systems, might be associated with covid-19
Recent evidence has indicated that mortality and readmission after discharge are common in individuals admitted to hospital with covid-19, but the long term epidemiology of multiorgan morbidity has not been quantified

**What this study adds**

Individuals discharged from hospital after acute covid-19 had increased rates of multiorgan dysfunction (particularly respiratory and cardiometabolic) compared with a matched control group from the general population
The rate ratio of multiorgan dysfunction (comparing individuals with covid-19 and matched controls) after discharge was greater in those aged less than 70 than in those aged 70 or more, and in ethnic minority groups than in the white population
Our findings suggest that the diagnosis, treatment, and prevention of post-covid syndrome requires integrated rather than organ or disease specific approaches

## Web extra

Extra material supplied by authors

Web appendix: Code lists for outcome variables

Web appendix: Supplementary tables

Web appendix: Sensitivity analyses

## Notes

Contributors: DA, KK, VN, BH, and AB conceptualised and designed the study. DA and TM prepared the study data and performed the statistical analysis. All authors contributed to interpretation of the results. KK, VN, BH, ID, and AB contributed to critical revision of the manuscript. All authors approved the final manuscript. DA is the guarantor for the study. The corresponding author attests that all listed authors meet authorship criteria and that no others meeting the criteria have been omitted.

Ethical approval: NHS Digital approved access to the data for the study after a favourable recommendation from its Independent Group Advising on the Release of Data. Ethical approval was obtained from the National Statistician’s Data Ethics Advisory Committee (NSDEC(20)12).

Data sharing: In accordance with NHS Digital’s Information Governance requirements, the study data cannot be shared.

The manuscript’s guarantor affirms that this manuscript is an honest, accurate, and transparent account of the study being reported; that no important aspects of the study have been omitted; and that any discrepancies from the study as planned (and, if relevant, registered) have been explained.

Dissemination to participants and related patient and public communities: The size of the study population precludes direct dissemination to participants.

Provenance and peer review: Not commissioned; externally peer reviewed.
