# Supplementary material for: Post-covid syndrome in individuals admitted to hospital with covid-19: retrospective cohort study
Source: BMJ. 2021 Mar 31;372:n693. doi: 10.1136/bmj.n693 (PMC8010267; doi:10.1136/bmj.n693)
Supplement: Supplementary file 1 — Web appendix: Code lists for outcome variables [file ayod064273.ww1.pdf]

Supplementary Appendix: Code lists for outcome variables

1. ICD-10 codes for defining outcome variables from HES data

Condition	Codes
Heart failure	I50
Stroke	I60-64
Myocardial infarction	I21-22
Arrhythmia	I47-49
Respiratory illness	J00-99
Chronic kidney disease stages 3-5	N18.3-18.5, Z94.0, Z99.2
Chronic liver disease	K70-77
Type 1 diabetes mellitus	E10
Type 2 diabetes mellitus	E11

2. SNOMED codes for defining outcome variables from GDPPR data

GDPPR code lists.xlsx
