# Supplementary material for: Post-covid syndrome in individuals admitted to hospital with covid-19: retrospective cohort study
Source: BMJ. 2021 Mar 31;372:n693. doi: 10.1136/bmj.n693 (PMC8010267; doi:10.1136/bmj.n693)
Supplement: Supplementary file 2 — Web appendix: Supplementary tables [file ayod064273.ww2.pdf]

Supplementary Table 1. Description of variables used to match COVID-19 and control patients

**Variable**	**Categorisation**
Age group	<50 years, 50-69 years, ≥70 years
Sex	Female, male
Ethnic group	Asian, Black, White, Mixed/Other, Unknown
Region of residence	North (North East, North West, Yorkshire and the Humber), South (East of England, London, South East, South West), Midlands (East Midlands, West Midlands), Unknown
IMD quintile	1 (most deprived), 2, 3, 4, 5 (least deprived), Unknown
Smoking status	Current, Former, Never/Unknown
BMI group	<25 kg/m^2 or Unknown, 25 kg/m^2 to <30 kg/m^2, ≥30 kg/m^2
History of hypertension	Yes, No
History of MACE	Yes, No
History of respiratory disease	Yes, No
History of CKD stages 3-5	Yes, No
History of CLD	Yes, No
History of diabetes	Yes, No
History of cancer	Yes, No

Table notes: BMI: body mass index; CKD: chronic kidney disease; CLD: chronic liver disease; IMD: Index of Multiple Deprivation; MACE: major adverse cardiovascular event (a composite of heart failure, myocardial infarction, stroke, and arrhythmia).

Supplementary Table 2. Counts and rates of adverse events contrasting individuals with COVID-19 in England discharged from hospital by 31 August 2020 with matched controls

**Adverse event (sample size per group)**	**COVID-19 cases**		**Control group**	
	**Events (n, %)**	**Rate per 1,000 person-years (95% CI)**	**Events (n, %)**	**Rate per 1,000 person-years (95% CI)**
Death (*n* = 47,780)	5,875 (12.3%)	320.0 (311.9 to 328.3)	830 (1.7%)	41.3 (38.6 to 44.3)
Readmission to hospital (*n* = 47,780)	14,060 (29.4%)	766.0 (753.4 to 778.8)	4,385 (9.2%)	218.9 (212.4 to 225.4)
Respiratory disease (all events) (*n* = 47,780)	14,140 (29.6%)	770.5 (757.8 to 783.3)	2,585 (5.4%)	129.1 (124.2 to 134.2)
Respiratory disease (new onset) (*n* = 28,335)	6,085 (21.5%)	538.9 (525.5 to 552.6)	240 (0.8%)	19.7 (17.3 to 22.4)
Diabetes (all events) (*n* = 47,780)	2,330 (4.9%)	126.9 (121.8 to 132.2)	1,725 (3.6%)	86.0 (82.0 to 90.2)
Diabetes (new onset) (*n* = 36,100)	400 (1.1%)	28.7 (26.0 to 31.7)	125 (0.3%)	8.2 (6.9 to 9.8)
MACE (all events) (*n* = 47,780)	2,315 (4.8%)	126.1 (121.0 to 131.4)	855 (1.8%)	42.6 (39.8 to 45.5)
MACE (new onset) (*n* = 36,130)	945 (2.6%)	65.9 (61.8 to 70.3)	190 (0.5%)	12.3 (10.6 to 14.1)
CKD (all events) (*n* = 47,780)	710 (1.5%)	38.7 (35.9 to 41.6)	410 (0.9%)	20.4 (18.5 to 22.5)
CKD (new onset) (*n* = 41,705)	240 (0.6%)	14.6 (12.8 to 16.6)	125 (0.3%)	7.2 (6.0 to 8.5)
CLD (all events) (*n* = 47,780)	135 (0.3%)	7.2 (6.1 to 8.6)	50 (0.1%)	2.5 (1.9 to 3.3)
CLD (new onset) (*n* = 46,395)	70 (0.2%)	4.0 (3.2 to 5.1)	15 (0.04%)	0.9 (0.5 to 1.4)

Table notes: CI: confidence interval; CKD: chronic kidney disease stages 3-5; CLD: chronic liver disease; MACE: major adverse cardiovascular event. Adverse events calculated from hospital episodes to 31 August 2020, and primary care records and deaths registrations to 30 September 2020. 'Readmission to hospital' represents any admission after discharge for COVID-19 cases, and any post-index admission for controls. COVID-19 cases were matched to controls on baseline demographic characteristics (age, sex, ethnicity, region, Index of Multiple Deprivation quintile, smoking status) and clinical histories (hypertension, major adverse cardiovascular event, respiratory disease, chronic kidney disease, chronic liver disease, diabetes, cancer).

Supplementary Table 3. Counts and rates of adverse events contrasting individuals with COVID-19 in England discharged from hospital by 31 August 2020 with matched controls, stratified by ICU versus non-ICU admission

Group (sample size)	Adverse event	COVID-19 cases		Control group	
		Events (n, %)	Rate per 1,000 person-years (95% CI)	Events (n, %)	Rate per 1,000 person-years (95% CI)
ICU patients (*n* = 4,745)	Death	420 (8.8%)	237.9 (215.7 to 261.8)	20 (0.5%)	11.4 (7.2 to 17.3)
	Readmission	1,235 (26.0%)	701.2 (662.6 to 741.4)	260 (5.5%)	134.8 (118.9 to 152.2)
	Respiratory disease	1,620 (34.1%)	918.6 (874.4 to 964.5)	100 (2.1%)	53.1 (43.3 to 64.4)
	Diabetes	335 (7.1%)	190.2 (170.4 to 211.7)	175 (3.6%)	90.0 (77.1 to 104.5)
	MACE	175 (3.6%)	98.2 (84.1 to 114.0)	30 (0.6%)	15.1 (10.1 to 21.7)
	CKD	65 (1.3%)	35.8 (27.5 to 45.8)	20 (0.4%)	9.4 (5.6 to 14.8)
Non-ICU patients (*n* = 43,035)	Death	5,455 (12.7%)	328.8 (320.1 to 337.6)	805 (1.9%)	44.5 (41.5 to 47.7)
	Readmission	12,825 (29.8%)	772.9 (759.5 to 786.4)	4,125 (9.6%)	227.8 (220.9 to 234.8)
	Respiratory disease	12,525 (29.1%)	754.7 (741.6 to 768.1)	2,485 (5.8%)	137.2 (131.8 to 142.7)
	Diabetes	1,995 (4.6%)	120.2 (115.0 to 125.6)	1,550 (3.6%)	85.6 (81.4 to 90.0)
	MACE	2,140 (5.0%)	129.1 (123.7 to 134.7)	825 (1.9%)	45.5 (42.4 to 48.7)
	CKD	645 (1.5%)	39.0 (36.0 to 42.1)	390 (0.9%)	21.6 (19.5 to 23.8)

Table notes: CI: confidence interval; CKD: chronic kidney disease stages 3-5; ICU: intensive care unit; MACE: major adverse cardiovascular event. Adverse events calculated from hospital episodes to 31 August 2020, and primary care records and deaths registrations to 30 September 2020. 'Readmission' represents any admission after discharge for COVID-19 cases, and any post-index admission for controls. COVID-19 cases were matched to controls on baseline demographic characteristics (age, sex, ethnicity, region, Index of Multiple Deprivation quintile, smoking status) and clinical histories (hypertension, major adverse cardiovascular event, respiratory disease, chronic kidney disease, chronic liver disease, diabetes, cancer).

Supplementary Table 4. Rates and rate ratios of adverse events contrasting individuals with COVID-19 in England discharged from hospital by 31 August 2020 with matched controls, stratified by demographic factors

**Group (sample size)**	**Adverse event**	**Rate per 1,000 person-years (95% CI)**		**Rate ratio (95% CI)**
		**COVID-19 cases**	**Control group**	
Sex: Male (*n* = 26,245)	Death	318.4 (307.5 to 329.6)	38.5 (34.9 to 42.3)	8.3 (7.5 to 9.2)
	Readmission	757.5 (740.7 to 774.7)	214.3 (205.8 to 223.1)	3.5 (3.4 to 3.7)
	Respiratory	780.9 (763.8 to 798.3)	127.3 (120.7 to 134.1)	6.1 (5.8 to 6.5)
	Diabetes	142.4 (135.2 to 150.0)	95.6 (90.0 to 101.6)	1.5 (1.4 to 1.6)
	MACE	130.7 (123.7 to 137.9)	43.0 (39.2 to 47.1)	3.0 (2.7 to 3.4)
	CKD	43.7 (39.7 to 48.0)	20.5 (17.9 to 23.4)	2.1 (1.8 to 2.5)
Sex: Female (*n* = 21,535)	Death	322.0 (309.9 to 334.5)	44.8 (40.6 to 49.4)	7.2 (6.5 to 8.0)
	Readmission	776.4 (757.5 to 795.7)	224.5 (214.8 to 234.5)	3.5 (3.3 to 3.6)
	Respiratory	757.6 (738.9 to 776.6)	131.4 (124.0 to 139.1)	5.8 (5.4 to 6.1)
	Diabetes	107.9 (101.0 to 115.3)	74.2 (68.6 to 80.0)	1.5 (1.3 to 1.6)
	MACE	120.6 (113.2 to 128.3)	42.0 (37.9 to 46.5)	2.9 (2.5 to 3.2)
	CKD	32.5 (28.8 to 36.7)	20.3 (17.5 to 23.5)	1.6 (1.3 to 1.9)
Age group: <70 years (*n* = 25,955)	Death	86.2 (80.7 to 91.9)	6.1 (4.7 to 7.7)	14.1 (11.0 to 18.3)
	Readmission	556.6 (542.6 to 570.8)	127.0 (120.5 to 133.8)	4.4 (4.1 to 4.6)
	Respiratory	615.8 (601.1 to 630.8)	58.5 (54.1 to 63.1)	10.5 (9.7 to 11.4)
	Diabetes	123.9 (117.4 to 130.7)	73.2 (68.2 to 78.4)	1.7 (1.6 to 1.8)
	MACE	55.7 (51.4 to 60.4)	13.0 (11.0 to 15.3)	4.3 (3.6 to 5.2)
	CKD	24.4 (21.6 to 27.5)	6.9 (5.4 to 8.6)	3.5 (2.7 to 4.6)
Age group: ≥70 years (*n* = 21,825)	Death	658.4 (640.1 to 677.0)	85.6 (79.6 to 91.9)	7.7 (7.1 to 8.3)
	Readmission	1,069.0 (1,045.7 to 1,092.6)	334.2 (322.3 to 346.5)	3.2 (3.1 to 3.3)
	Respiratory	994.2 (971.7 to 1,017.0)	217.8 (208.2 to 227.7)	4.6 (4.3 to 4.8)
	Diabetes	131.3 (123.2 to 139.8)	102.1 (95.6 to 109.0)	1.3 (1.2 to 1.4)
	MACE	227.9 (217.3 to 239.0)	79.7 (74.0 to 85.8)	2.9 (2.6 to 3.1)
	CKD	59.3 (53.9 to 65.1)	37.4 (33.5 to 41.6)	1.6 (1.4 to 1.8)
Ethnic group: White (*n* = 34,355)	Death	403.4 (392.5 to 414.5)	53.1 (49.4 to 57.0)	7.6 (7.0 to 8.2)
	Readmission	876.8 (860.7 to 893.2)	263.6 (255.3 to 272.2)	3.3 (3.2 to 3.5)
	Respiratory	861.1 (845.1 to 877.3)	164.5 (157.9 to 171.3)	5.2 (5.0 to 5.5)
	Diabetes	119.6 (113.6 to 125.7)	85.5 (80.8 to 90.4)	1.4 (1.3 to 1.5)
	MACE	153.4 (146.7 to 160.3)	53.9 (50.2 to 57.9)	2.8 (2.6 to 3.1)
Ethnic group: Non-White (*n* = 8,315)	Death	126.6 (115.0 to 139.0)	12.7 (9.3 to 16.9)	10.0 (7.3 to 13.8)
	Readmission	553.7 (529.2 to 579.0)	126.1 (114.8 to 138.2)	4.4 (4.0 to 4.9)
	Respiratory	567.8 (543.0 to 593.4)	49.9 (42.9 to 57.8)	11.4 (9.8 to 13.3)
	Diabetes	184.1 (170.1 to 198.9)	121.1 (110.1 to 133.0)	1.5 (1.3 to 1.7)
	MACE	68.2 (59.8 to 77.4)	18.8 (14.6 to 23.8)	3.6 (2.8 to 4.8)

Table notes: CI: confidence interval; CKD: chronic kidney disease stages 3-5; MACE: major adverse cardiovascular event. Adverse events calculated from hospital episodes to 31 August 2020, and primary care records and deaths registrations to 30 September 2020. 'Readmission' represents any admission after discharge for COVID-19 cases, and any post-index admission for controls. COVID-19 cases were matched to controls on baseline demographic characteristics (age, sex, ethnicity, region, Index of Multiple Deprivation quintile, smoking status) and clinical histories (hypertension, major adverse cardiovascular event, respiratory disease, chronic kidney disease, chronic liver disease, diabetes, cancer). Rates and rate ratios for CKD could not be stratified by ethnic group due to insufficient event counts in the control group.
