# Supplementary material for: Post-covid syndrome in individuals admitted to hospital with covid-19: retrospective cohort study
Source: BMJ. 2021 Mar 31;372:n693. doi: 10.1136/bmj.n693 (PMC8010267; doi:10.1136/bmj.n693)
Supplement: Supplementary file 3 — Web appendix: Sensitivity analyses [file ayod064273.ww3.pdf]

Supplementary Appendix: Sensitivity analyses

1. Comparison of matched and unmatched COVID-19 cases, and the impact of excluding unmatched cases from the main analysis

Of 86,955 individuals in hospital with COVID-19 during the study period, 53,795 (61.9%) had been discharged alive by end-of-study. After excluding individuals with unknown age or sex and those who could not be matched to a control, 47,780 COVID-19 cases were included in the analysis, representing 90.8% of those discharged alive with known age and sex. Compared with those in the matched population, the 9.2% of individuals who remained unmatched were more likely to belong to an ethnic minority group, live in a deprived area, currently smoke, have been admitted to hospital during the baseline period, and have comorbidities (Table 1).

Table 1. Baseline characteristics of matched and unmatched COVID-19 cases

Characteristic	Category	Matched cases (*n* = 47,780)	Unmatched cases (*n* = 4,865)	P-value	Standardised difference (%)
Age	<30 years	2,255 (4.7%)	135 (2.8%)	<0.0001	10.1
	30-49 years	7,760 (16.2%)	1,020 (20.9%)	<0.0001	-12.1
	50-69 years	15,945 (33.4%)	1,700 (34.9%)	0.0292	-3.3
	≥70 years	21,825 (45.7%)	2,010 (41.3%)	<0.0001	8.8
Sex	Male	26,245 (54.9%)	2,540 (52.3%)	0.0004	5.3
	Female	21,535 (45.1%)	2,325 (47.7%)	0.0004	-5.3
Ethnicity	White	34,355 (71.9%)	2,115 (43.5%)	<0.0001	60.1
	Asian	4,320 (9.0%)	975 (20.1%)	<0.0001	-31.7
	Black	2,565 (5.4%)	865 (17.8%)	<0.0001	-39.5
	Mixed/Other	1,430 (3.0%)	545 (11.2%)	<0.0001	-32.3
	Unknown	5,110 (10.7%)	365 (7.5%)	<0.0001	11.2
Index of Multiple Deprivation quintile	1 (most deprived)	11,510 (24.1%)	1,435 (29.5%)	<0.0001	-12.3
	2	10,970 (23.0%)	1,270 (26.1%)	<0.0001	-7.4
	3	9,265 (19.4%)	850 (17.5%)	0.0015	4.9
	4	8,315 (17.4%)	710 (14.6%)	<0.0001	7.7
	5 (least deprived)	7,695 (16.1%)	535 (11.0%)	<0.0001	15.1
	Unknown	25 (<0.1%)	65 (1.3%)	<0.0001	-15.3
Smoking status	Current	4,000 (8.4%)	685 (14.1%)	<0.0001	-18.1
	Former	19,560 (40.9%)	1,730 (35.6%)	<0.0001	10.9
	Never	20,295 (42.5%)	2,095 (43.1%)	0.4301	-1.2
	Unknown	3,920 (8.2%)	355 (7.2%)	0.0175	3.7
Body mass index	<25 kg/m^2	9,415 (19.7%)	1,050 (21.6%)	0.0019	-4.6
	25 to <30 kg/m^2	12,140 (25.4%)	1,165 (23.9%)	0.0248	3.4
	≥30 kg/m^2	15,390 (32.2%)	1,745 (35.9%)	<0.0001	-7.7
	Unknown	10,835 (22.7%)	905 (18.6%)	<0.0001	10.1
Previous hospitalisation		39,575 (82.8%)	4,720 (97.1%)	<0.0001	-48.8
Hypertension		24,720 (51.7%)	3,440 (70.7%)	<0.0001	-39.7
Respiratory disease		19,440 (40.7%)	3,325 (68.4%)	<0.0001	-57.9
Asthma		8,695 (18.2%)	1,320 (27.2%)	<0.0001	-21.6
COPD		6,565 (13.7%)	915 (18.8%)	<0.0001	-13.7
Other		11,890 (24.9%)	2,285 (47.0%)	<0.0001	-47.3
Diabetes		11,680 (24.4%)	2,770 (57.0%)	<0.0001	-70.2
Type 1		1,235 (2.6%)	490 (10.1%)	<0.0001	-31.1
Type 2		11,530 (24.1%)	2,710 (55.7%)	<0.0001	-68.2
Major adverse cardiovascular event		11,650 (24.4%)	2,370 (48.8%)	<0.0001	-52.4
Heart failure		5,255 (11.0%)	1,190 (24.5%)	<0.0001	-35.9
Stroke		3,040 (6.4%)	640 (13.1%)	<0.0001	-23.0
Myocardial infarction		2,265 (4.7%)	530 (10.9%)	<0.0001	-23.0
Arrhythmia		7,170 (15.0%)	1,180 (24.3%)	<0.0001	-23.6
Cancer		9,820 (20.5%)	1,930 (39.7%)	<0.0001	-42.7
Chronic kidney disease stages 3-5		6,075 (12.7%)	1,830 (37.7%)	<0.0001	-60.0
Dementia		5,010 (10.5%)	540 (11.1%)	0.1580	-2.1
Osteoporosis		4,390 (9.2%)	530 (10.9%)	0.0001	-5.8
Rheumatoid arthritis		1,890 (4.0%)	265 (5.5%)	<0.0001	-7.1
Chronic liver disease		1,380 (2.9%)	1,440 (29.6%)	<0.0001	-77.8

Taken together, the aforementioned differences in characteristics between the matched and unmatched populations mean that the outcome rates presented in the main analysis, which are based on only the matched population, may slightly underestimate the rates in the full population of discharged COVID-19 cases (Table 2). The estimates presented in the main results may therefore be considered conservative.

Table 2. Counts and rates of death, readmission, and multi-organ dysfunction comparing matched and all COVID-19 cases

**Outcome**	**Matched COVID-19 cases (*n* = 47,780)**		**All COVID-19 cases (*n* = 52,645)**	
	**Events (n, %)**	**Rate per 1,000 person-years (95% CI)**	**Events (n, %)**	**Rate per 1,000 person-years (95% CI)**
Death	5,875 (12.3%)	320.0 (311.9 to 328.3)	6,555 (12.5%)	324.7 (316.9 to 332.6)
Readmission to hospital	14,060 (29.4%)	766.0 (753.4 to 778.8)	16,065 (30.5%)	795.5 (783.2 to 807.9)
Respiratory disease (all events)	14,140 (29.6%)	770.5 (757.8 to 783.3)	15,910 (30.2%)	787.8 (775.6 to 800.1)
Respiratory disease (new onset)	6,085 (21.5%)	538.9 (525.5 to 552.6)	6,435 (21.5%)	541.0 (527.8 to 554.4)
Diabetes (all events)	2,330 (4.9%)	126.9 (121.8 to 132.2)	2,805 (5.3%)	138.8 (133.7 to 144.0)
Diabetes (new onset)	400 (1.1%)	28.7 (26.0 to 31.7)	425 (1.1%)	28.9 (26.2 to 31.8)
MACE (all events)	2,315 (4.8%)	126.1 (121.0 to 131.4)	2,655 (5.0%)	131.4 (126.5 to 136.5)
MACE (new onset)	945 (2.6%)	65.9 (61.8 to 70.3)	1,030 (2.7%)	67.2 (63.1 to 71.4)
CKD (all events)	710 (1.5%)	38.7 (35.9 to 41.6)	1,055 (2.0%)	52.3 (49.2 to 55.6)
CKD (new onset)	240 (0.6%)	14.6 (12.8 to 16.6)	270 (0.6%)	15.6 (13.8 to 17.5)
CLD (all events)	135 (0.3%)	7.2 (6.1 to 8.6)	205 (0.4%)	10.1 (8.7 to 11.5)
CLD (new onset)	70 (0.2%)	4.0 (3.2 to 5.1)	80 (0.2%)	4.1 (3.3 to 5.1)

Table notes: CI: confidence interval; CKD: chronic kidney disease stages 3-5; CLD: chronic liver disease; MACE: major adverse cardiovascular event. Outcomes calculated from hospital episodes to 31 August 2020, and primary care records and deaths registrations to 30 September 2020. COVID-19 cases were matched to controls on baseline demographic characteristics (age, sex, ethnicity, region, Index of Multiple Deprivation quintile, smoking status) and clinical histories (hypertension, major adverse cardiovascular event, respiratory disease, chronic kidney disease, chronic liver disease, diabetes, cancer).

2. Impact of conducting regression adjustment after matching

We investigated the possibility of residual confounding by age after matching, since we had to use coarse age groups to ensure a sufficient match rate. The age distributions were well balanced between COVID-19 cases and controls within all three matching groups, with mean ages of: 36.2 and 36.7, respectively, in the <50 years group; 59.4 and 59.4, respectively, in the 50-69 years group; and 81.4 and 79.4, respectively, in the ≥70 years group. The proportion of individuals in the <30 years age group was slightly greater among COVID-19 cases (4.7%) than in the matched control group (2.5%).

We also found imbalance between COVID-19 cases and controls in terms of unknown smoking status (8.2% and 4.6%, respectively) and BMI (22.7% and 16.5%, respectively). As with age, this imbalance was due to use of coarsened versions of the smoking status and BMI variables during matching, in which individuals with unknown smoking status were grouped with non-smokers, and individuals with unknown BMI were grouped with those with BMI <25 kg/m^2.

We therefore assessed the robustness of our main results by adjusting for a second-order polynomial of age and non-coarsened versions of smoking status and BMI (each including a separate 'Unknown' category) in a Poisson regression of outcome counts, including the natural logarithm of person-years as an offset term. This analysis revealed very little change in the estimated rate ratios between cases and controls (Figure 1), including when the analysis was stratified by demographic characteristics (Figure 2), indicating the absence of residual confounding by age, smoking status, or BMI after matching.

Figure 1. Rate ratios comparing COVID-19 cases with matched controls, with and without regression adjustment for age, smoking status, and BMI

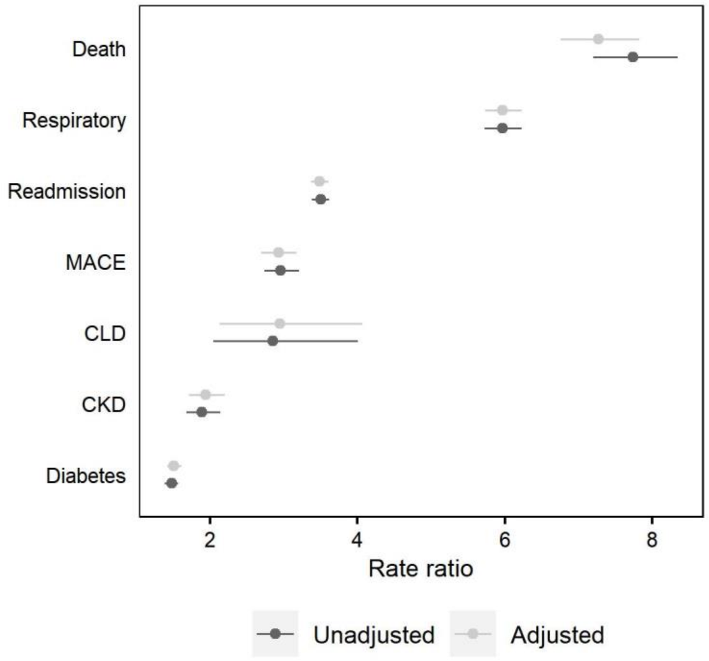

Figure notes: CKD: chronic kidney disease; CLD: chronic liver disease; MACE: major adverse cardiovascular event. Outcomes calculated from hospital episodes to 31 August 2020, and primary care records and deaths registrations to 30 September 2020. ''Readmission to hospital' represents any admission after discharge for COVID-19 cases, and any post-index admission for controls. COVID-19 cases were matched to controls on baseline demographic characteristics (age, sex, ethnicity, region, Index of Multiple Deprivation quintile, smoking status) and clinical histories (hypertension, MACE, respiratory disease, CKD, CLD, diabetes, cancer). Adjusted estimates were obtained from a Poisson regression of outcome counts on group (case or control), a second-order polynomial of age, smoking status, and BMI category, including log-exposure as an offset term.

Figure 2. Rate ratios comparing COVID-19 cases with matched controls, with and without regression adjustment for age, smoking status, and BMI, stratified by demographic factors

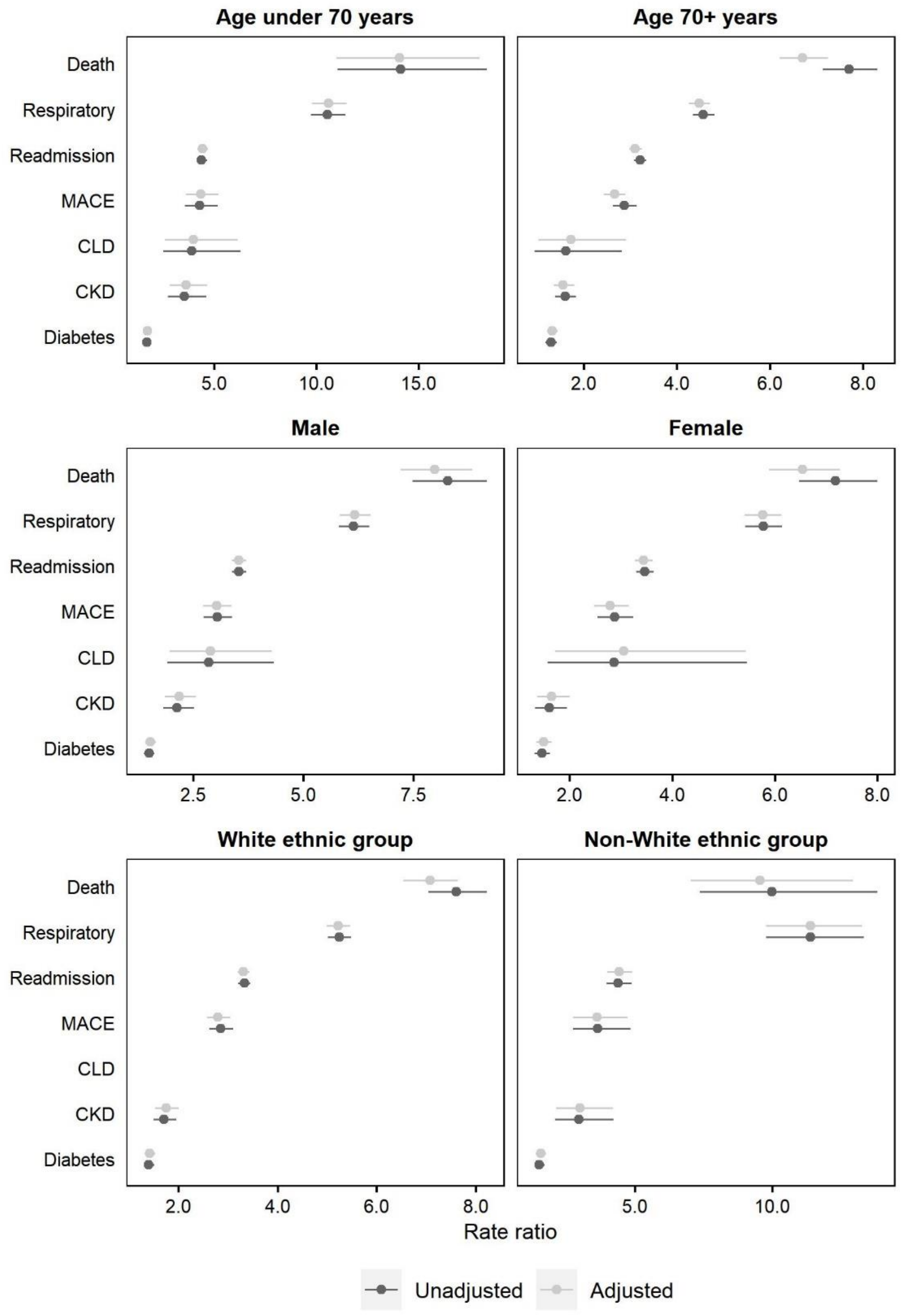

Figure notes: CKD: chronic kidney disease; MACE: major adverse cardiovascular event. Outcomes calculated from hospital episodes to 31 August 2020, and primary care records and deaths registrations to 30 September 2020. 'Readmission to hospital' represents any admission after discharge for COVID-19 cases, and any post-index admission for controls. Individuals with missing ethnicity information were omitted from the analysis stratified by ethnic group.COVID-19 cases were matched to controls on baseline demographic characteristics (age, sex, ethnicity, region, Index of Multiple Deprivation quintile, smoking status) and clinical histories (hypertension, MACE, respiratory disease, CKD, CLD, diabetes, cancer). Adjusted estimates were obtained from a Poisson regression of outcome counts on group (case or control), a second-order polynomial of age, smoking status, and BMI category, including log-exposure as an offset term. Rate ratios for CKD could not be stratified by ethnic group due to insufficient event counts in the control group.

3. Impact of including only laboratory-confirmed cases of COVID-19 in the study

Individuals with COVID-19 were included in the study if they had a hospital episode starting from 1 January 2020 and ending by 31 August 2020 with a primary diagnosis of COVID-19, identified using International Statistical Classification of Diseases and Related Health Problems 10th Revision (ICD-10) codes U07.1 (virus identified) and U07.2 (virus not identified); that is, by a positive laboratory test or clinical diagnosis. We included cases coded to U07.2 to recognise that not all patients with COVID-19 would have received a test during their hospital episode, particularly during the early weeks of the pandemic, and we wanted to recognise the importance of professional judgement on the part of clinicians. In sensitivity analysis, we restricted the case definition to diagnoses coded to U07.1 only (representing 80.2% of COVID-19 cases included in the main analysis), finding that comparisons between event rates of cases and controls were robust to this restriction (Table 3).

Table 3. Counts and rates of death, readmission, and multi-organ dysfunction comparing laboratory-confirmed COVID-19 cases with matched controls

**Outcome**	**COVID-19 cases**		**Control group**	
	**Events (n, %)**	**Rate per 1,000 person-years (95% CI)**	**Events (n, %)**	**Rate per 1,000 person-years (95% CI)**
Death	4,935 (12.9%)	334.1 (324.8 to 343.5)	705 (1.8%)	43.5 (40.4 to 46.9)
Readmission to hospital	11,135 (29.1%)	753.8 (739.8 to 767.9)	3,615 (9.4%)	223.0 (215.8 to 230.4)
Respiratory disease (all events)	10,820 (28.2%)	732.6 (718.9 to 746.6)	2,120 (5.5%)	130.9 (125.4 to 136.6)
Respiratory disease (new onset)	4,710 (20.7%)	517.8 (503.1 to 532.8)	205 (0.9%)	21.0 (18.2 to 24.1)
Diabetes (all events)	1,965 (5.1%)	133.1 (127.3 to 139.1)	1,435 (3.8%)	88.7 (84.2 to 93.4)
Diabetes (new onset)	330 (1.2%)	29.7 (26.6 to 33.1)	105 (0.4%)	8.5 (6.9 to 10.3)
MACE (all events)	1,810 (4.7%)	122.5 (117.0 to 128.3)	685 (1.8%)	42.3 (39.2 to 45.6)
MACE (new onset)	725 (2.5%)	63.0 (58.5 to 67.8)	150 (0.5%)	12.2 (10.3 to 14.3)
CKD (all events)	590 (1.5%)	39.9 (36.8 to 43.3)	345 (0.9%)	21.2 (19.1 to 23.6)
CKD (new onset)	195 (0.6%)	15.1 (13.0 to 17.3)	105 (0.3%)	7.4 (6.0 to 8.9)
CLD (all events)	105 (0.3%)	7.2 (5.9 to 8.7)	35 (0.1%)	2.3 (1.6 to 3.2)
CLD (new onset)	60 (0.2%)	4.3 (3.3 to 5.5)	15 (<0.1%)	0.8 (0.4 to 1.4)

Table notes: CI: confidence interval; CKD: chronic kidney disease stages 3-5; CLD: chronic liver disease; MACE: major adverse cardiovascular event. Outcomes calculated from hospital episodes to 31 August 2020, and primary care records and deaths registrations to 30 September 2020. ''Readmission to hospital' represents any admission after discharge for COVID-19 cases, and any post-index admission for controls. COVID-19 cases were matched to controls on baseline demographic characteristics (age, sex, ethnicity, region, Index of Multiple Deprivation quintile, smoking status) and clinical histories (hypertension, major adverse cardiovascular event, respiratory disease, chronic kidney disease, chronic liver disease, diabetes, cancer). Laboratory-confirmed cases of COVID-19 were those with a primary diagnosis code of U07.1 during the hospital spell.
